# Preliminary study of time maximum intensity projection computed tomography imaging for the detection of early ischemic change in patient with acute ischemic stroke

**DOI:** 10.1097/MD.0000000000009906

**Published:** 2018-03-02

**Authors:** Kazuhiro Murayama, Shigetaka Suzuki, Ryo Matsukiyo, Akinori Takenaka, Motoharu Hayakawa, Takashi Tsutsumi, Kenji Fujii, Kazuhiro Katada, Hiroshi Toyama

**Affiliations:** a Department of Radiology, Fujita Health University; b Department of Neurosurgery, Fujita Health University, Toyoake; c Clinical Application Research Center, Toshiba Medical Systems Corporation, Otawara; d Joint Research Laboratory of Advanced Medical Imaging, Fujita Health University, Toyoake, Japan.

**Keywords:** CT angiography source image, early ischemic change, time maximum intensity projection CT technique

## Abstract

Noncontrast computed tomography (NCCT) has been used for the detection of early ischemic change (EIC); however, correct interpretation of NCCT findings requires much clinical experience. This study aimed to assess the accuracy of time maximum intensity projection computed tomography technique (tMIP), which reflects the maximum value for the time phase direction from the dynamic volume data for each projected plane, for detection of EIC, against that of NCCT.

Retrospective review of NCCT, cerebral blood volume in CT perfusion (CTP-CBV), and tMIP of 186 lesions from 280 regions evaluated by Alberta Stroke Program Early CT Score (ASPECTS) in 14 patients with acute middle cerebral artery stroke who had undergone whole-brain CTP using 320-row area detector CT was performed. Four radiologists reviewed EIC on NCCT, CTP-CBV, and tMIP in each ASPECTS region at onset using the continuous certainty factor method. Receiver operating characteristic analysis was performed to compare the relative performance for detection of EIC. The correlations were evaluated.

tMIP-color showed the best discriminative value for detection of EIC. There were significant differences in the area under the curve for NCCT and tMIP-color, CTP-CBV (*P* < .05). Scatter plots of ASPECTS showed a positive significant correlation between NCCT, tMIP-gray, tMIP-color, and the follow-up study (NCCT, *r* = 0.32, *P* = .0166; tMIP-gray, *r* = 0.44, *P* = .0007; tMIP-color, *r* = 0.34, *P* = .0104).

Because tMIP provides a high contrast parenchymal image with anatomical and vascular information in 1 sequential scan, it showed greater accuracy for detection of EIC and predicted the final infarct extent more accurately than NCCT based on ASPECTS.

## 1 Introduction

Evaluation of early ischemic change (EIC) on noncontrast computed tomography (NCCT) in patients with acute ischemic stroke is important to determine the need for tissue plasminogen activator therapy.^[1–3]^ However, there is a considerable lack of agreement on the recognition and quantification of early changes during CT examination.^[4]^ Therefore, more experience is required for correct evaluation of EIC. CT angiography source image (CTA-SI),^[5–9]^ CT perfusion source image (CTP-SI),^[10]^ and cerebral blood volume on CTP (CTP-CBV)^[11]^ may facilitate the evaluation of EIC. CTA-SI provides a measure of cerebral blood flow,^[12]^ and by delineation of avascular areas, it can predict the final infarct extent more accurately than NCCT.^[5,7,8]^ CTP-CBV was found more useful than CTA-SI for detection of middle cerebral artery (MCA) acute stroke in a retrospective cohort of patients who were imaged within 3 h from stroke onset.^[11]^ Because the decrease in area of CTP-CBV corresponds to ischemic core, CTP-CBV is useful in the detection of EICs and to guide treatment decision making in patients with acute ischemic stroke.^[11]^ Based on the evidence from large-scale studies, CTP-CBV has been frequently used in recent years to assess the ischemic core and to assess the need for thrombolytic therapy.^[13]^

Dynamic volume data by the area detector CT were used as the time averaging data in the only arterial or venous phase.^[14]^ Time maximum intensity projection computed tomography imaging (tMIP) reflects the maximum value image in every projected plane from the time-phase direction on all timepoints from CTP acquisition. Because we can expect improvement in the signal-to-noise ratio (SNR) in tMIP as compared to that with conventional time averaging, it is expected to increase the contrast between brain gray matter and white matter. In addition, detection of the ischemic area in tMIP is more convenient than that with NCCT or CTA-SI individually, as the CTA-SI and the CT venography (CTV) source images are added to the brain parenchyma in tMIP. Sakai et al^[15]^ demonstrated that maximum intensity projection (MIP) images provide more precise information on the distribution of micronodules in lung diseases and may help radiologists in training to differentiate between lung diseases. Gruden et al^[16]^ also reported that MIP processing reduced the number of overlooked small lung nodules. Based on the advantages of MIP images in lung diseases and the advantages of CTA-SI mentioned above, we hypothesized that the increased SNR of gray matter and white matter in tMIP, which is a combination of MIP images and CTA-SI, may be superior to NCCT for detection of EIC. However, robust evidence of the advantages of tMIP over CTA-SI, CTP-CBV, and NCCT is yet to be obtained.

In this study, we evaluated the performance of tMIP for detection of EIC in patients with acute cerebrovascular disease. The objective was to assess accuracy of tMIP technique for detection of EIC with that of CTP-CBV and NCCT.

## 2 Methods

### 2.1 Subjects

Our institutional review board approved this opt-out research design. Opt-out opportunities were provided to all participants included in this retrospective study. NCCT findings of 24 consecutive patients with acute MCA stroke within 24 h after the onset of symptoms between October 2013 and September 2014 were included. Images from 10 patients were excluded either because of the time interval between NCCT and CTP, motion artifact, development of hemorrhagic complication, small white matter lacunar infarcts, or because the scanning parameters were different from that defined in the protocol. Therefore, 14 patients (7 men and 7 women; age range, 66–93 years; mean age, 75.2 years) who underwent whole-brain CTP using 320-row area detector CT within 24 h of the NCCT scan at admission and NCCT or diffusion weighted image (DWI) as the follow-up study during 0 to 13 days (mean: 4 days) were selected (Table 1). Of the 280 regions evaluated by Alberta Stroke Program Early CT Score (ASPECTS),^[17,18]^ 186 lesions were detected.

**Table 1:**
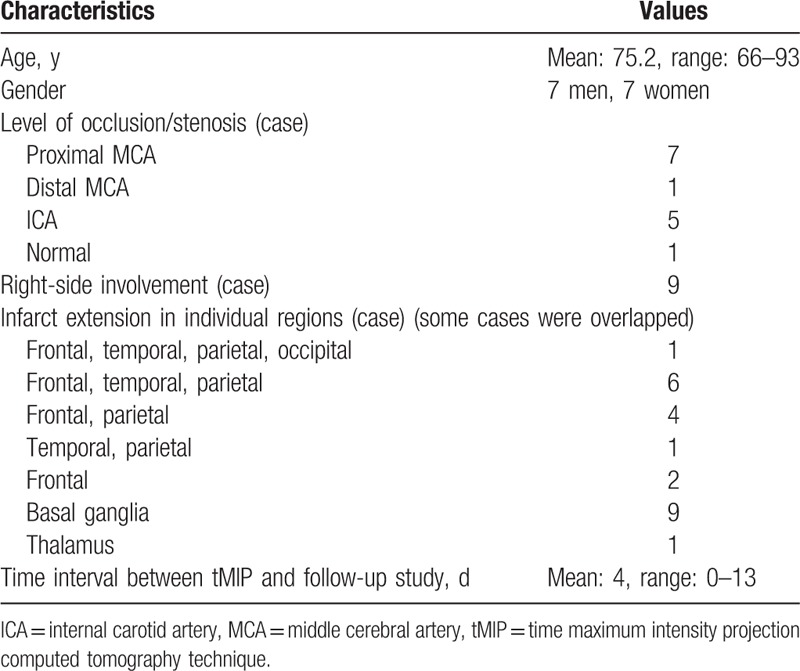
Clinical characteristics of 14 patients with acute middle cerebral artery stroke.

### 2.2 CT and MRI protocol

Dynamic volume data for CTP were acquired using a 320-row area detector CT scanner (Aquilion ONE; Toshiba Medical Systems Corporation, Otawara, Japan) with a scan range of 160 mm (320 slices × 0.5 mm), tube voltage of 80 kV, tube current of 70 mA in the arterial phase and 30 mA in the venous phase, and a gantry rotation speed of 1 s/rotation. A tube voltage of 120 kV was used only for 2 patients. Dynamic volume data were acquired by intermittent scanning (1 s in the arterial phase and 5 s in the venous phase). Contrast medium volume was calculated based on the patient's body weight, and 250 mg I/kg of Iopamidol (Iopamiron 370; Bayer, Osaka, Japan) was injected intravenously as a bolus at a fixed 10 s, followed by an intravenous bolus injection of 30 mL of physiological saline solution at the same rate as that of the contrast medium.

DWI was performed using 1.5T magnetic resonance imaging (MRI) scanner (Achieva 1.5T Nova Dual with an 8-channel brain coil; Philips Healthcare, Best, The Netherlands), 3T MRI scanner (Ingenia 3.0T with dS Head coil; Philips Healthcare, Best, The Netherlands), or 3T MRI scanner (Vantage Titan 3T with a 16- or 32-channel coil; Toshiba Medical Systems Corporation, Otawara, Japan). The following pulse sequence parameters were used: echo planar imaging spin echo, time to repeat/echo time = 4000 ms/73 ms, matrix = 128 × 153, echo planar imaging factor = 67, bandwidth = 16.9 Hz, sensitivity encoding parallel imaging = 2.4, slice thickness = 5 mm, slice gap = 1 mm, and scan time = 52.0 s.

NCCT was acquired using 80- or 320-row CT scanner (Aquilion Prime, Aquilion ONE; Toshiba Medical Systems Corporation) with a scan range of 160 mm (320 slices × 0.5 mm), tube voltage 120 kV, auto exposure control = standard deviation 3.7, pitch factor 0.64, and a gantry rotation speed of 0.75 s/rotation.

### 2.3 Image postprocessing

We calculated the CTP-CBV axial maps of 5 mm thickness using the analysis software installed in the CT console (Aquilion ONE, 4D Perfusion; Toshiba Medical Systems Corporation). The CTP analysis algorithm used was the reformulated singular value decomposition (SVD+) method based on block-circulant SVD (bSVD), which is unaffected by tracer delay effects.^[19,20]^ The arterial input function was set in the internal carotid artery on the unaffected side.

**Figure 1:**
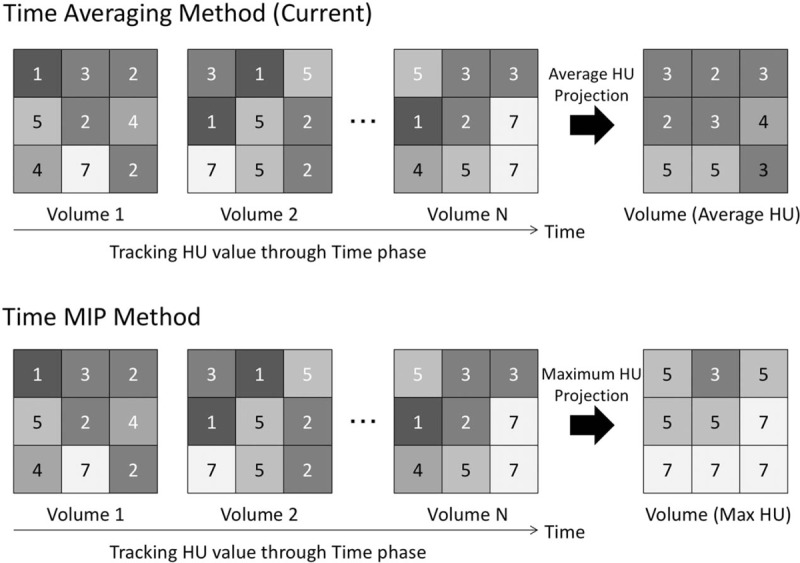
The difference of time-averaging method and time maximum intensity projection method. Average of each voxel data in the entire volume data in current time-averaging method are reflected, whereas maximum values are reflected in time maximum intensity projection method. HU = hounsfield unit, MIP = maximum intensity projection.

The dynamic data using the area detector CT were treated using the time average imaging procedure to add the data for the phase along temporal axes at any consecutive phases so far.^[14]^ Conversely, tMIP image reflected the maximum value of each matrix in the dynamic data for all time phases (Fig. 1). We calculated the tMIP images using the analysis software installed in the CT console. The tMIP images were generated from the entire dynamic data acquired for CTP. tMIP gray scale (tMIP-gray) and tMIP color scale (tMIP-color) were made by a 5 mm thickness. Brain perfusion color scale equipped with CT device console was used.

### 2.4 Data analysis

Presence or absence of EIC in each ASPECTS region was determined on NCCT, CTP-CBV, tMIP-gray, and tMIP-color performed at the time of onset using the continuous certainty factor method. Two board-certified radiologists (KM, 12 years of experience; SS, 12 years of experience) and 2 resident radiologists (AT: 3 years of experience; RM: 3 years of experience) participated as observers. They were blinded to the number of positive images and performed ASPECTS evaluation independently at random with patient's clinical information such as chief complaints. The final infarct cores were determined on DWI or on follow-up NCCT.

Receiver operating characteristic (ROC) curve analysis was used to calculate the sensitivity, specificity, and diagnostic accuracy of onset NCCT, CTP-CBV, tMIP-gray, tMIP-color, and DWI (or follow-up NCCT). Correlation between onset NCCT, CTP-CBV, tMIP-gray, tMIP-color and DWI (or follow-up NCCT) was evaluated for each ASPECT region.

### 2.5 Statistical analysis

Area under the curve (AUC) from ROC analysis was compared between NCCT and others for determining the best parameter with the highest AUC. Chi-squared test was used to calculate *P* values for comparisons of AUC. For all statistical analyses, *P* values of ≤.05 were considered indicative of a statistically significant between-group difference. Spearman rank correlation analysis was used to calculate correlation coefficients and *P* values for comparisons of findings of NCCT, CTP-CBV, tMIP, and follow-up DWI or NCCT for each ASPECT region. Statistical analysis was performed using commercially available statistical software (Graph Pad PRISM, version 6, San Diego, CA; BellCurve for Excel, version 2.11, Social Survey Research Information Co, Ltd. Tokyo, Japan).

## 3 Results

**Figure 2:**
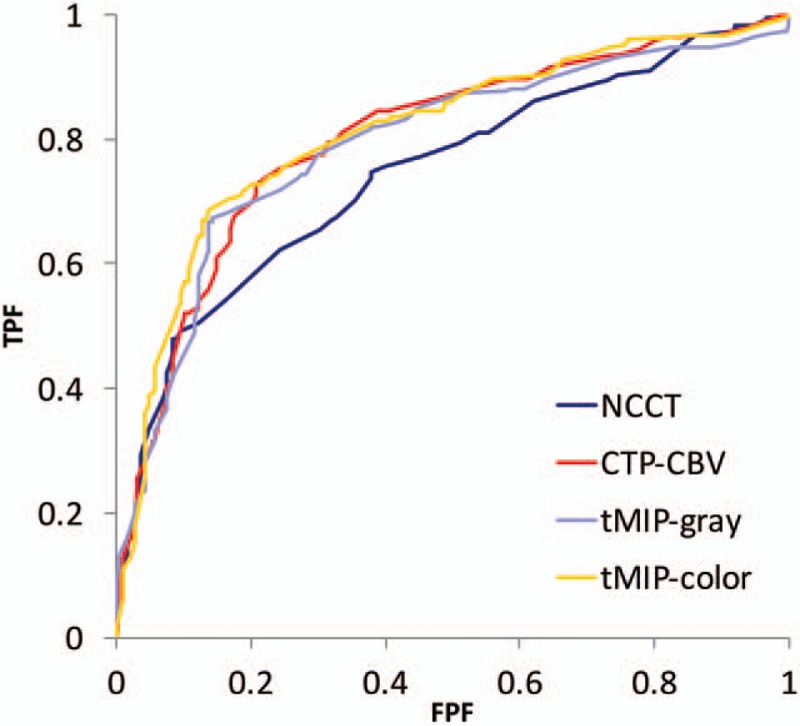
Receiver-operating characteristic curve analysis for evaluation of early ischemic changes. (A) NCCT; (B) CTP-CBV; (C) tMIP-gray; and (D) tMIP-color. tMIP-color showed the best discriminative value in the evaluation of early ischemic changes. CBV = cerebral blood volume, CTP = CT perfusion, FPF = false positive fraction, NCCT = noncontrast computed tomography, tMIP = time maximum intensity projection CT technique, TPF = true positive fraction.

The results of the ROC analysis are summarized in Table 2 and Fig. 2. tMIP-color had the best discriminative value for detection of EIC (AUC, 0.811; sensitivity, 71.5%; specificity, 81.4%) compared with that of CTP-CBV (AUC, 0.801; sensitivity, 72.6%; specificity, 79.3%), tMIP-gray (AUC, 0.790; sensitivity, 68.0%; specificity, 84.0%), and NCCT (AUC, 0.745; sensitivity, 62.1%; specificity, 75.5%). There were significant differences in the AUC between NCCT and tMIP-color, CTP-CBV (*P* < .05). On the other hand, there was no significant difference in the AUC between NCCT and tMIP-gray. There was no significant difference with respect to assessment of the tMIP-color scale between the 2 board-certified radiologists and the 2 resident radiologists; however, the AUC based on the assessment of board-certified radiologists (0.8339) tended to be greater (*P* = .0775) than the AUC based on the assessment of the resident radiologists (0.7851). There was no significant difference in the assessment of NCCT (AUC: 0.7382 vs. 0.7562, *P* = .6363), CTP-CBV (AUC: 0.8019 vs. 0.8018, *P* = .9961), and tMIP-gray (AUC: 0.7930 vs. 0.7829, *P* = .7562) between board-certified radiologists and resident radiologists, respectively (Table 3).

**Table 2:**
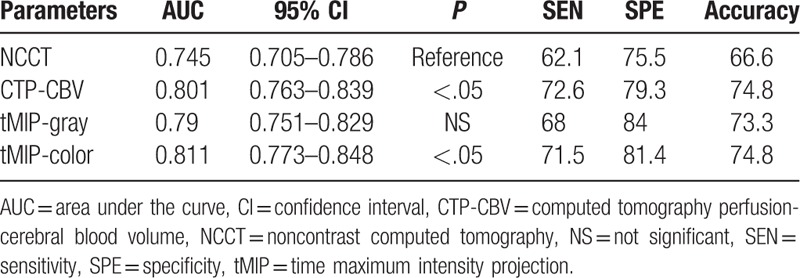
Receiver-operating characteristics curve analysis of NCCT, CTP-CBV, tMIP-gray, and tMIP-color for detection of early ischemic change.

**Table 3:**
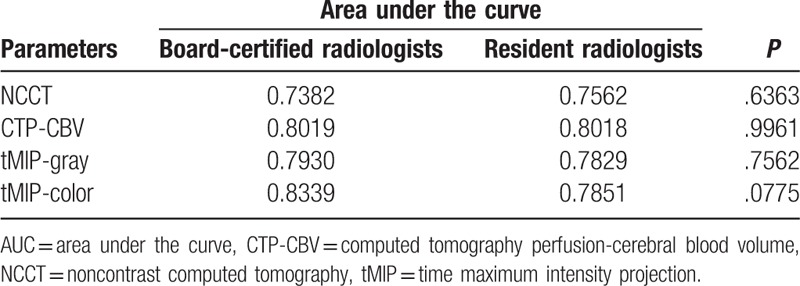
Comparison of AUC values between board-certified radiologists and resident radiologists.

**Figure 3:**
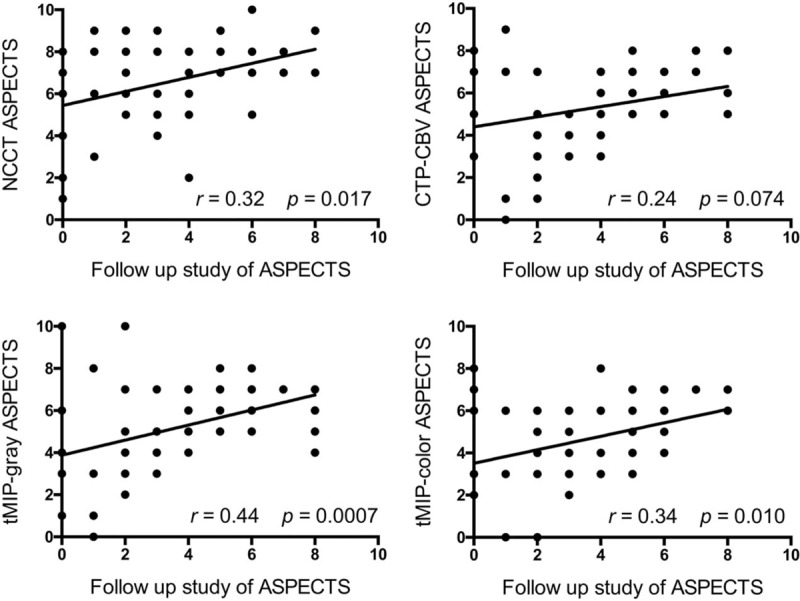
Comparison of ASPECTS based on NCCT, CTP-CBV, tMIP-gray, and tMIP-color. All patients were included in all 4 comparisons (all 56 results for 14 patients, 4 observers were plotted; some scores were overlapped on the graph). Scatter plots of ASPECTS showed a positive significant correlation between NCCT, tMIP-gray, tMIP-color, and the follow-up study (*P* < .05). Scatter plots of ASPECTS showed a positive correlation between CTP-CBV and the follow-up study, but the difference was not statistically significant (*P* > .05). “The follow-up study of ASPECTS” was defined as the ASPECTS scored by NCCT or diffusion weighted image performed as the follow-up study during 0 to 13 days (mean: 4 days) after the onset. ASPECTS = Alberta Stroke Program Early CT Score, CBV = cerebral blood volume, CTP = CT perfusion, NCCT = noncontrast computed tomography, tMIP = time maximum intensity projection CT technique.

Scatter plots of ASPECTS showed a positive significant correlation between NCCT, tMIP-gray, tMIP-color, and the follow-up study (NCCT; *r* = 0.32, *P* = .0166; tMIP-gray; *r* = 0.44, *P* = .0007; tMIP-color; *r* = 0.34, *P* = .0104) (Fig. 3). Scatter plots of ASPECTS showed a positive correlation between CTP-CBV and the follow-up study, but the difference was not statistically significant (*r* = 0.24, *P* = .0738) (Fig. 3).

## 4 Discussion

The tMIP technique not only enhances the gray matter/white matter contrast but also delineates any ischemic area due to the enhancement of avascular area. Therefore, tMIP is more useful than NCCT for the detection of EIC. tMIP provides anatomical information, and ASPECTS evaluation with tMIP can be performed more precisely than with CTP-CBV.

**Figure 4:**
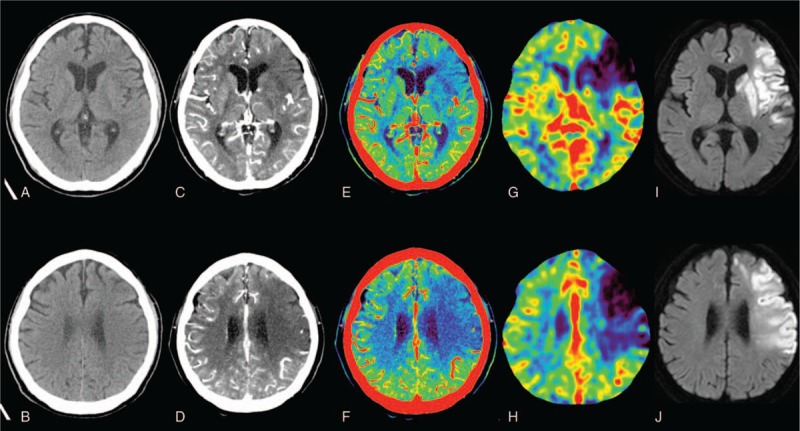
Onset NCCT (A, B), tMIP-gray (C, D), tMIP-color (E, F), CTP-CBV (G, H), and follow-up DWI (I, J) in representative patients with acute cerebral infarction caused by left ICA occlusion. This NCCT image is almost normal. However, the tMIP-gray and tMIP-color images clearly show low attenuation and avascular area in the left frontal-parietal lobe and basal ganglia, which correspond to low CTP-CBV areas. Follow-up DWI showed low-intensity area in the same location in which the onset NCCT shows EIC. CBV = cerebral blood volume, CTP = CT perfusion, DWI = diffusion weighted image, EIC = early ischemic change, ICA = internal carotid artery, NCCT = noncontrast computed tomography, tMIP = time maximum intensity projection CT technique.

The tMIP-color showed a significantly larger AUC than NCCT in ROC analysis. Color scale of TMI images improves diagnostic accuracy on visual evaluation; therefore, tMIP-color was found to be more useful for detection of EIC than NCCT in this study (Fig. 4). Camargo et al^[5]^ reported that CT angiographic source image is more sensitive in detection of early irreversible ischemia and more accurate for prediction of the final infarct volume. tMIP is a new technique that incorporates both CTA-SI (for detection of ischemic penumbra) and NCCT (for detection of the ischemic core) and makes it easier to identify ischemic area. In a study aimed at identification of infarct core and penumbra in acute stroke using CTP-SIs, Wang et al^[10]^ reported that CTP source imaging (CTP-SI) during the arterial phase and venous phase mismatch model could possibly be applied to penumbra and infarct core. Therefore, tMIP reflects the maximum value of all arteriovenous data and shows ischemic penumbra, including the ischemic core.

Our findings regarding the usefulness of CTP-CBV show good agreement with those of previous studies^[21]^ in which reduction in CBV was shown to indicate ischemic core as measured by using ASPECTS. CTP-CBV also showed a significantly larger AUC than NCCT in ROC analysis. However, CTP-CBV did not show a significant positive relationship with follow-up DWI or NCCT as compared with that shown by tMIP. This was because CTP-CBV did not provide more anatomical information than tMIP. tMIP images provide anatomical information, and tMIP can predict the final infarct extent more precisely than NCCT and CTP-CBV when assessed using ASPECTS criteria. Concomitant use of both tMIP and CTP-CBV does not provide any additional leverage in the detection of EIC. In addition, Pulli et al^[8]^ reported that infarct core estimation on CTA-SIs depends on CTA parameters such as scan speed, acquisition direction (from C1 vertebra to vertex or from vertex to aortic arch) due to the use of helical scans. However, this is not necessarily a problem of these scan protocols,^[8,22]^ and the evaluation range can cover whole brain because 320-row area detector CT that we used for tMIP does not require helical scan. In addition, because tMIP was not CTP, which needs a complicated analysis algorithm, it was unaffected by differences among commercial software for analysis,^[23]^ and analysis error of the basic principles of SVD method.^[24]^ The tMIP appears to be a much simpler method than CTP for the detection of EIC.

MIP method was claimed to be useful for the education of residents in chest imaging.^[15]^ In our study, there was no significant difference in the AUC for tMIP and other imaging modalities between board-certified radiologists and resident radiologists; however, in the case of residents, the AUC for tMIP tended to be greater than that for NCCT. Therefore, use of tMIP may improve the ability of beginners, including that of the resident radiologists, to detect lesions.

This study had several limitations. First, variability in color scales between different models may affect the tMIP images. Because tMIP is not a cerebral blood flow map, it is not clear as to which reference table should be used. Further, standardization of the color scale may be required as in the CTP map. Second, the number of subjects was small in this retrospective analysis. Future prospective studies should include more subjects. Third, the motion correction technique is not employed in tMIP. Fourth, the effects of streak artifacts in areas such as the middle cranial fossa and posterior cranial fossa are likely to be more severe in tMIP images. Fifth, use of 2 different kinds of voltage for scanning may affect the image appearance.

In conclusion, the tMIP technique is superior for detection of the EIC due to the enhanced contrast between gray matter and white matter. The defect of the enhancement effect of CTP-SI is equivalent to EIC. Because tMIP provides high-contrast parenchymal image as well as vascular and anatomical information in 1 sequential scan, it showed greater accuracy than that of NCCT for detection of EIC and predicted the final infarct extent more precisely, when assessed based on ASPECTS criteria.
